# The state of One Health research across disciplines and sectors – a bibliometric analysis

**DOI:** 10.1016/j.onehlt.2020.100146

**Published:** 2020-06-06

**Authors:** Sarah Humboldt-Dachroeden, Olivier Rubin, Snorre Sylvester Frid-Nielsen

**Affiliations:** Department of Social Science and Business, Roskilde University, Roskilde, Denmark

**Keywords:** One Health, Bibliometric analysis, Network analysis, Interdisciplinary studies, Implementation, Management

## Abstract

There is a growing interest in One Health, reflected by the rising number of publications relating to One Health literature, but also through zoonotic disease outbreaks becoming more frequent, such as Ebola, Zika virus and COVID-19.

This paper uses bibliometric analysis to explore the state of One Health in academic literature, to visualise the characteristics and trends within the field through a network analysis of citation patterns and bibliographic links. The analysis focuses on publication trends, co-citation network of scientific journals, co-citation network of authors, and co-occurrence of keywords.

The bibliometric analysis showed an increasing interest for One Health in academic research. However, it revealed some thematic and disciplinary shortcomings, in particular with respect to the inclusion of environmental themes and social science insights pertaining to the implementation of One Health policies. The analysis indicated that there is a need for more applicable approaches to strengthen intersectoral collaboration and knowledge sharing. Silos between the disciplines of human medicine, veterinary medicine and environment still persist. Engaging researchers with different expertise and disciplinary backgrounds will facilitate a more comprehensive perspective where the human-animal-environment interface is not researched as separate entities but as a coherent whole. Further, journals dedicated to One Health or interdisciplinary research provide scholars the possibility to publish multifaceted research. These journals are uniquely positioned to bridge between fields, strengthen interdisciplinary research and create room for social science approaches alongside of medical and natural sciences.

## 1 Introduction

One Health joins the three interdependent sectors – animal health, human health, and ecosystems – with the goal to holistically address health issues such as zoonotic diseases, antimicrobial resistance, food-borne diseases and environmental conditions [1]. In 2010, the Food and Agriculture Organization (FAO), the World Organization for Animal Health (OIE) and the World Health Organization (WHO) engaged in a tripartite collaboration to ensure a multisectoral perspective to effectively manage and coordinate a One Health approach. One Health is defined as.

*“an approach to address a health threat at the human-animal-environment interface based on collaboration, communication, and coordination across all relevant sectors and disciplines, with the ultimate goal of achieving optimal health outcomes for both people and animals; a One Health approach is applicable at the subnational, national, regional, and global level”* [2].

This paper uses bibliometric analysis to explore the state of One Health in academic literature, to visualise the characteristics and trends within the field through a network analysis of citation patterns and bibliographic links. A bibliometric analysis is a quantitative method to capture, in this case, the networks of journals, authors and occurrences of keywords. By investigating these citation indices, it is possible to get an overview of the academic features and dynamics, the strengths and the shortcomings, that characterise a particular scientific field [3]. Previous bibliometric studies have investigated the use of One Health documents, examining journals over time, tracking the increase of public health research involving animals, or investigating the issue of citation indices in relation to veterinary medicine and One Health publications [[4], [5], [6]]. This paper is the first to use bibliometric analysis to explore One Health contributions across disciplines and sectors.

## 2 Methodology

**Fig. 1:**
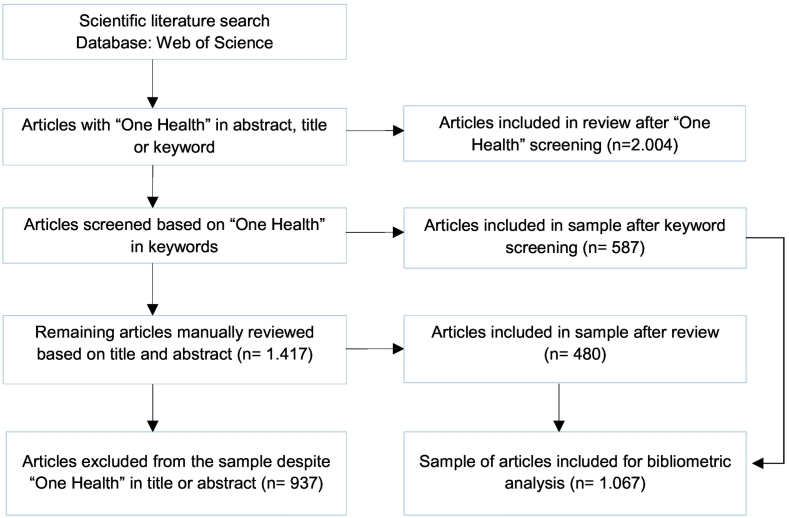
Flow chart of literature search in the Web of Science database.

The data for the bibliometric analysis is drawn from the Web of Science (WoS). The WoS is arguably one of the largest academic multidisciplinary databases, and it contains more than 66.9 million contributions from the natural sciences (Science Citation Index Expanded), social sciences (Social Sciences Citation Index) and humanities (Arts & Humanities Citation Index) [7]. The broad scope of the database aligns well with the One Health concept's cross-disciplinary approach. The analytical period is demarcated by the first One Health publication included in the WoS in 1998 and it ends in December 2019. The search term “One Health” was applied to compile the first crude sample of articles that mention the concept of One Health in their title, keywords or abstract. This search, however, excluded articles that thematically address One Health, but do not label it as such, as well as articles that refer to the human-animal-environment interface before the term of One Health first appeared in 2004 [8]. With the applied method, some articles might have been excluded that address One Health. However, the analysis focused on the use of the One Health concept. Hence, articles that did not mention One Health but thematically address its inherent topics were not intended to be included. For the literature search, the basic assumption was that articles conducting One Health research (whether conceptually, methodologically and/or empirically) would as a minimum have mentioned “One Health” in the abstract, title or keywords. The literature search resulted in 2004 English articles, see flow chart in Fig. 1. However, this sample also included a sizable group of articles that just made use of “one health” in a sentence such as “one health district” or “one health professional”. To restrict the sample to contributions only pertaining to the *concept* of One Health, two subsequent screening measures were taken. First, 587 contributions which used One Health as a keyword were automatically included in the sample. Second, the abstract of the remaining contributions (1417) were manually screened to determine whether One Health was included as a concept or was just a generic syntax. This screening exercise led to 937 contributions being discarded. The final sample consisted of 1067 contributions pertaining to the *concept* of One Health.

The bibliometric analysis was conducted with the bibliometrix package for the R programming language. The analysis focuses on: 1) publication trends, 2) co-citation network of scientific journals, 3) co-citation network of authors, and 4) co-occurrence of keywords.

The publication trend is outlined using both absolute and relative number of One Health publications. The co-citation networks of scientific journals provide information on the disciplinary structure of the field of One Health while the co-citation network of authors disaggregates further to the citation patterns of individual authors. The co-citation network of journals shows the relation between the publications within the outlets. For example, when a publication within journal A cites publications within journals B and C, it indicates that journals B and C share similar characteristics. The more journals citing both B and C, the stronger their similarity. To minimise popularity bias among frequently cited journals, co-citation patterns are normalised through the Jaccard Index. The Jaccard Index measures the similarity between journals B and C as the intersection of journals citing both B and C, divided by the total number of journals that cited B and C individually [9,10]. Like the co-citation network of journals, the co-citation network for authors measures the similarity of authors in terms of how often they are cited by other authors, also normalised through the Jaccard Index. When author A cites both authors B and C, it signifies that B and C share similar characteristics.

The study also investigates the co-occurrence of keywords to identify the content of One Health publications. Here, co-occurrence measures the similarity of keywords based on the number of times they occur together in different articles. It provides information on the main other topical keywords linked to One Health and can thus be used to gauge the knowledge structure of the field. Here, the articles Keywords Plus are the unit of analysis. WoS automatically generates Keywords Plus based on the words or phrases appearing most frequently in an articles bibliography. Keywords Plus are more fruitful for bibliometric analyses than author keywords, as they convey more general themes, methods and research techniques [11].

Disciplinary clusters within the networks, illustrated by the colours in Fig. 3, Fig. 4, Fig. 5, are identified empirically applying the Louvain clustering algorithm [12]. Louvain is a hierarchical clustering algorithm that attempts to maximise modularity, measured by the density of edges between nodes within communities and sparsity between nodes across communities. The nodes represent the aggregated citations of the academic journals and the edges, the line between two nodes, display the relation between the journals. The shorter the path between the nodes the stronger their relation. Node size indicates “betweenness centrality” in the network, which is a measure of the number of shortest paths passing through each node [13]. Betweenness centrality estimates the importance of a node on the flow of information through the network, based on the assumption that information generally flows through the most direct communicative pathways.

## 3 Results

### 3.1 One Health publication trends

**Fig. 2:**
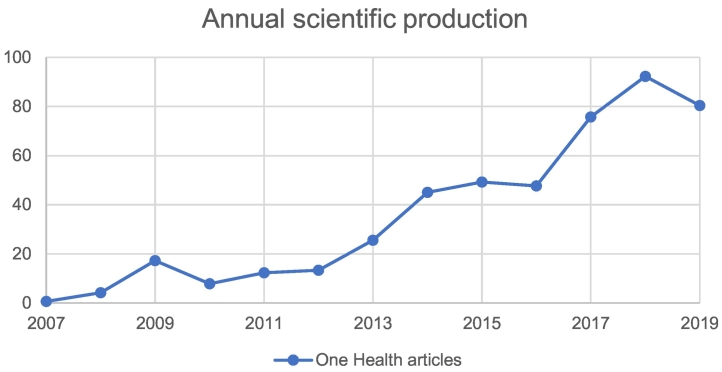
Ratio of annual scientific production of One Health articles.

In the period from 1998 to 2019, One Health publications have increased in both absolute and relative terms. The absolute number of publications referring to the concept of One Health has risen from one publication in the 1990s, to 39 in the 2000s, to 1027 in the 2010s. Especially in the 2010s, the annual number of publications rose steadily, passing 100 publications in 2015 and 200 in 2018. The relative number of publications, where we control for the general increase in academic publications in the WoS, reveals a similar pattern of increasing academic attention to the concept (Fig. 2). For every million publications in the three main WoS citation indices, 80 publications in 2019 mentioned the concept of One Health in their title, keywords or abstract. The annual scientific production has steadily increased after the initiation of the FAO/OiE/WHO collaboration in 2010 [14]. After 2016 in particular, the publications appear to have bourgeoned, which is consistent with the timelines of the Ebola and Zika virus outbreaks [15,16]. For example, the One Health publications in our sample relating to Ebola have more than tripled after 2016. One might, therefore, expect to observe a similar spike in One Health publications that study the COVID-19 outbreak in 2020.

### 3.2 Co-citation network of scientific journals

While the use of the One Health concept has increased, the co-citation network shows that the increase is mostly driven by the sectors of human and veterinary medicine, evidenced by their centrality in terms of information flows within the network.

**Fig. 3:**
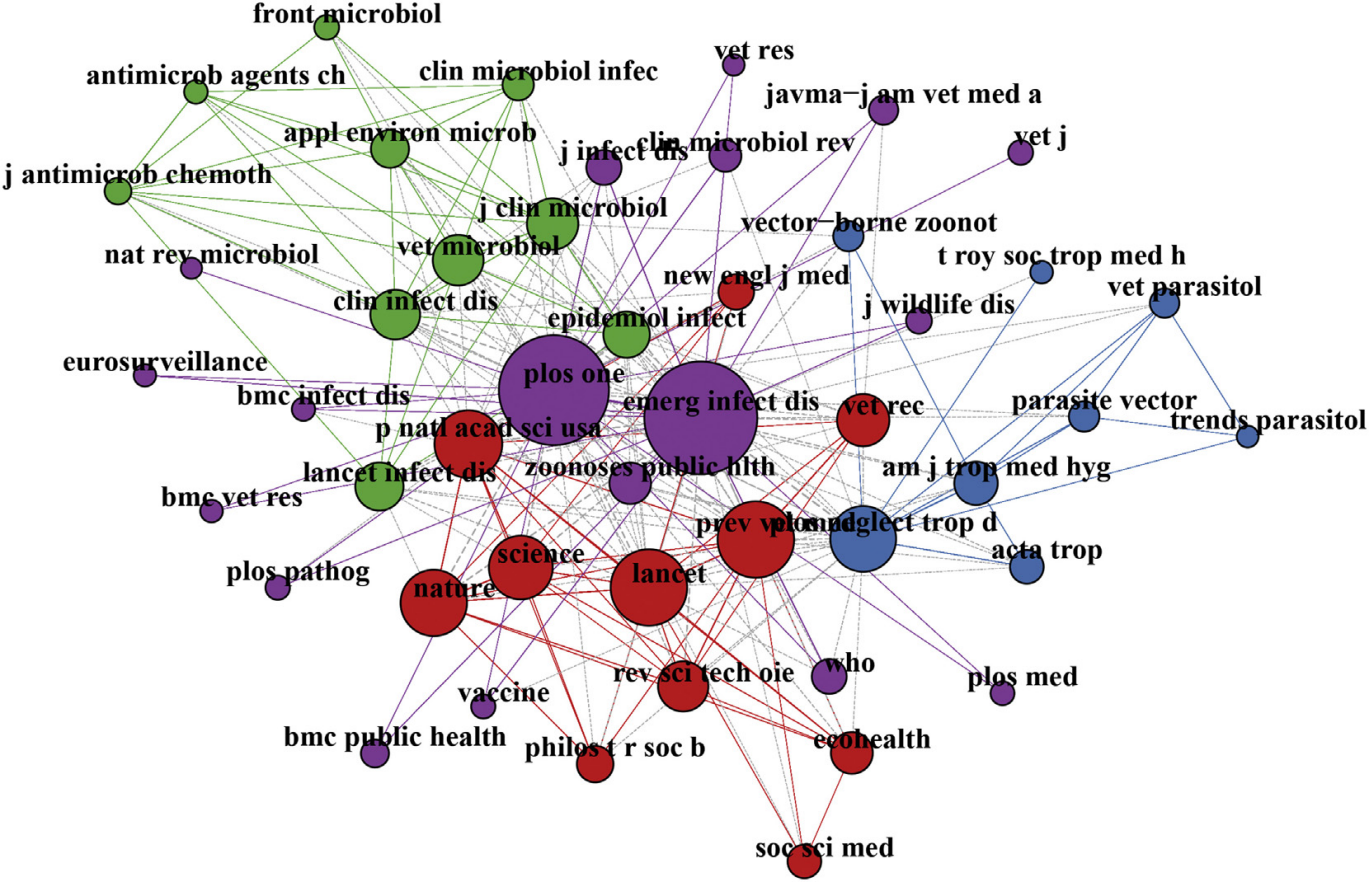
Co-citation network of scientific journals for One Health.

Fig. 3 visualises the co-citation network of journals, demonstrating four colour coded clusters. The clusters display journals which are most similar in terms of their co-citation patterns, which indicates specific disciplinary traits in the network. Since the clusters emerge inductively through the use of the Louvain algorithm, the meaning of the clusters was investigated qualitatively to allocate categories based on their shared characteristics. As a result, four main disciplinary groupings were identified (green: microbiology; blue: parasitology; purple: infectious diseases; red: general sciences). Most journals are in the field of infectious diseases, with *Plos One* and *Emerging Infectious Disease* as the most central outlets. The nodes *Plos One* and *Emerging Infectious Disease* have a high betweenness centrality which indicates a high level of influences on the flow of information throughout the network. The journals are heavily co-cited and connect to outlets covering all four areas. The cluster of the general sciences shows many co-citations links, especially within the same area, for example *Preventive Veterinary Medicine, The Lancet, Nature, Science* and the *Proceedings of the National Academy of Sciences of the United States of America.* However, there are also co-cited journals in the field of the general sciences, which indicate more social science contributions of One Health topics, including the journals *Social Science & Medicine* and *EcoHealth.* These journals allow for broader social and political science perspectives. The journals also show similar characteristics, as they are both co-cited with *The Lancet* and *Preventive Veterinary Medicine*. In the field of microbiology, the network shows strong interrelations between journals within the cluster and only modest relations to other clusters. The area of parasitology is also mostly co-cited in its own area. Here, most aggregated citations are rooted in the journal *PLOS Neglected Tropical Diseases*. In these last two clusters, microbiology and parasitology, the journals cover topics mainly exclusively pertaining to medical or biological sciences.

### 3.3 Co-citation network of authors

The most active One Health scholars, publishing more than ten articles over the last 12 years, are from the field of veterinary research. Of the top six researchers, five have a veterinary background (Jakob Zinsstag, Jonathan Rushton, Esther Schelling, Barbara Häsler and Bassirou Bonfoh). While Degeling is the only researcher of the top six with an education in the social sciences, the remaining five veterinarian scholars do touch upon social science themes within their publications, relating to systemic or conceptual approaches, sociopolitical dimensions and knowledge integration (e.g. Zinsstag and Schelling [17]; Häsler [18]; Rushton [19]. Five of the six most productive researchers work in Europe and three of them are associated with the same institute, namely the Swiss Tropical and Public Health Institute (Zinsstag, Schelling and Bonfoh) [20].There has been some cooperation across institutes and department as evidence by the co-authorships of Zinsstag and Häsler, Häsler and Rushton, Rushton and Zinsstag (e.g. [[21], [22], [23]]).

**Fig. 4:**
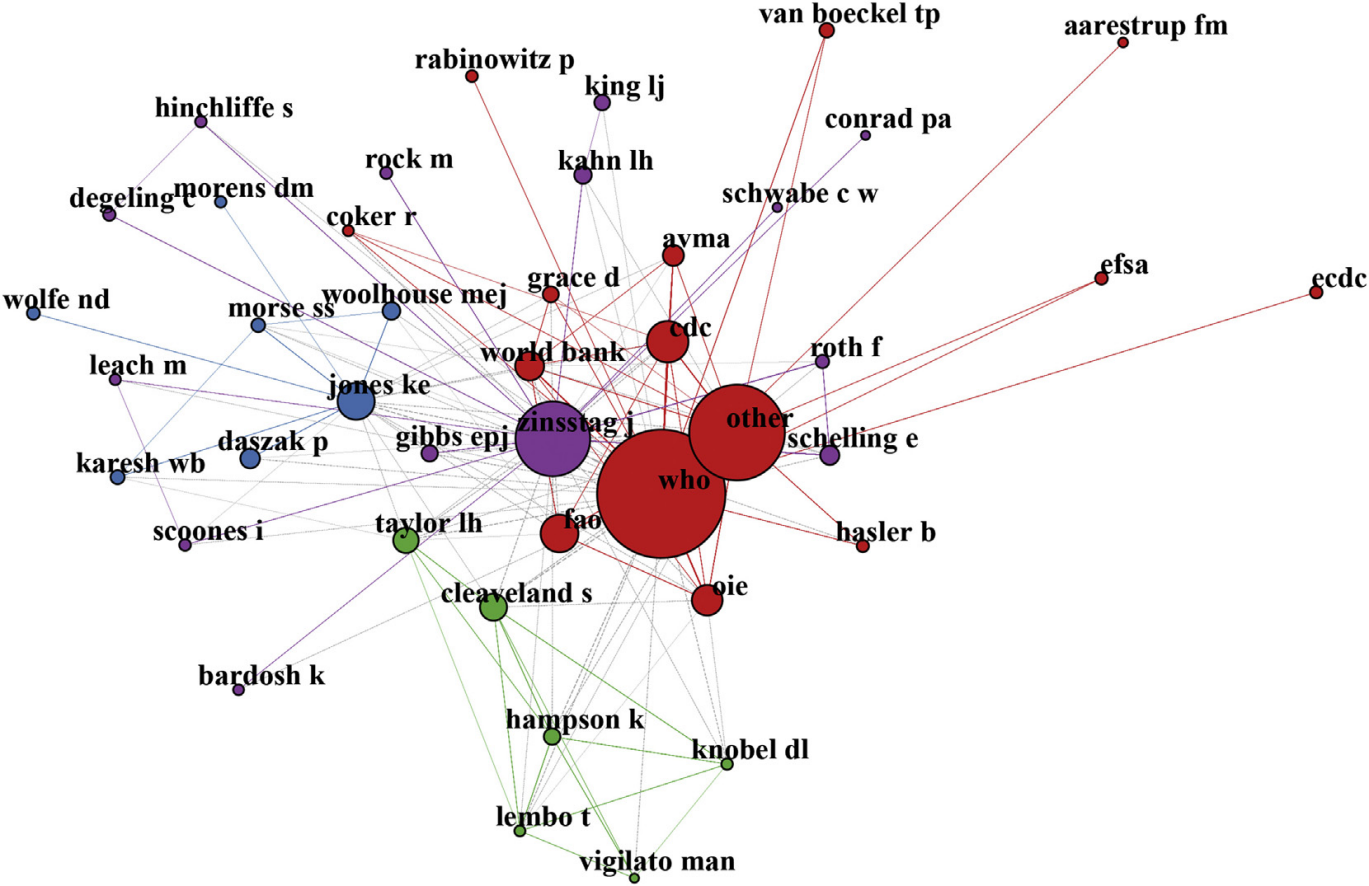
Co-citation network of authors working on One Health topics. The node “other” represents any publication without a specific author, which could be grey literature such as reports.

Fig. 4 illustrates the co-citation network of authors. Four clusters of authors emerged in the network (green: zoonoses and epidemiology; blue: biodiversity and ecohealth; purple: animal health, public health; red: policy-related disciplines). Academic scholars are mainly found in the green, blue and purple clusters, whereas the authors of the red clusters are mainly represented by organisations such as the WHO, CDC, FAO, OiE, and the World Bank. Generally, the network shows that the field is dominated by the WHO and Zinsstag (red and purple cluster), these clusters are also the most central to the flow of information in the network. Scholars within the biodiversity and ecohealth cluster are less connected with the other clusters but especially scholars in the green cluster are more isolated from the other clusters. More specific, the network shows that, Degeling and Steve Hinchliffe are often cited together with Zinsstag by other authors, indicating similar characteristics in their research. The network also shows that Zinsstag is often cited with the Schelling and Roth, which again signifies that the authors share similar properties. The organisations in the network are also co-cited by each other. Especially the WHO was co-cited heavily within the network. Notably, the European Centre for Disease Prevention and Control (ECDC) is not cited together with the American counterpart, the CDC. The European Food Safety Authority (EFSA) is an agency funded by the European Union that provides independent scientific advice on food safety, such as for animal and plant health, which embedded the One Health approach in its mission statement [24]. EFSA shows no direct connection to the ECDC or any other organisations. It indicates that the two European institutions are not commonly cited together in scientific publications despite covering similar One Health topics. However, EFSA and ECDC do collaborate, which for example results in the annual European Union reports on zoonoses and antimicrobial resistance [25,26].

### 3.4 Co-occurrences of keywords

**Fig. 5:**
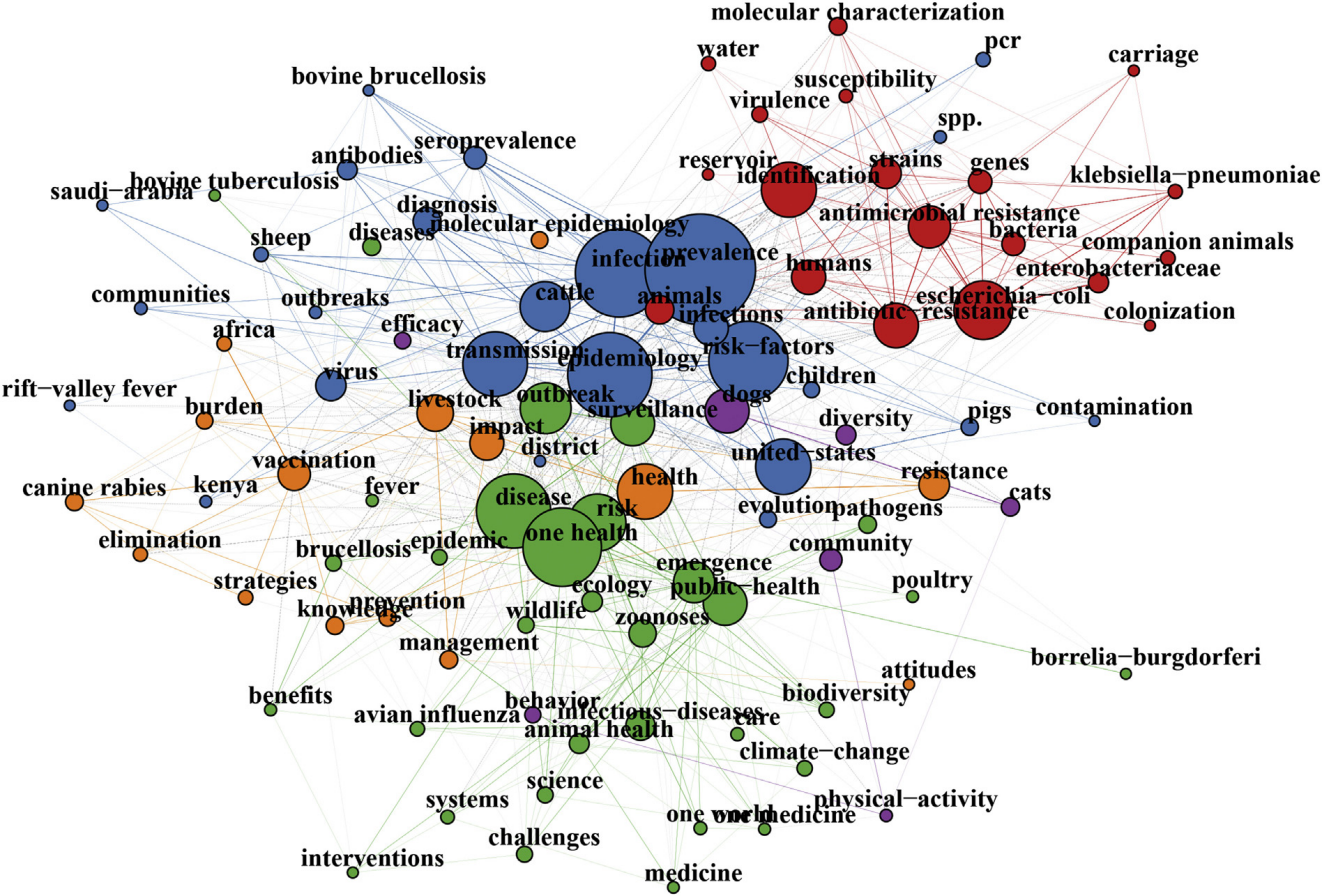
Co-occurrence of keywords within articles pertaining to One Health.

Within the bibliometric analysis, the co-occurrence of Keywords Plus in the reviewed articles is analysed to reveal topics and concepts that are the most mentioned and interconnected. The clustering algorithm produces five distinct thematic clusters within the network (red: microbiology; blue: medical science; green: veterinary and ecological science; orange: public health management; purple: anthropology). Fig. 5 illustrates that the blue ‘medical science’-cluster is very central, connecting to all other disciplines and showing strong relations within and outside the cluster. Most central keywords are prevalence, epidemiology, infection, risk factors and disease. The green cluster of veterinary and ecological science is also central in the network with many and strong links to the other clusters. The microbiology cluster is also a prominent cluster in the network, although it is primarily a self-referential cluster with limited keyword links to other thematic clusters. The public health management and anthropology clusters are the least prominent clusters. In the anthropology cluster, there are only seven nodes, which are sparsely distributed with distant connections and no central nodes. The orange cluster is more central but key concepts such as strategies, knowledge, management and attitudes play a peripheral role.

## 4 Discussion

There has been a steady increase of One Health articles, in particular in the wake of the FAO/OiE/WHO collaboration in 2010. External pressures primarily in the form of disease outbreaks such as Ebola and Zika virus also appear to have facilitated further research into One Health. In short, more and more scholars appear to display an interest in the holistic approach of One Health. While this is indeed a welcome development, the bibliometric analysis reveals certain shortcomings in the academic field of One Health that can be structured around three important dimensions: 1) diversity of sectors and disciplines; 2) themes and interfaces addressed; 3) scholars and institutions involved.

### 4.1 Sectors and disciplines

The citation network of journals showed that One Health is heavily researched in the sciences, particularly in the fields of microbiology, parasitology, infectious diseases and general sciences. The mostly cited journals for One Health themes are the epidemiological journal *Emerging Infectious Diseases* and the science journal *PLOS One*. This is certainly merited, as many One Health issues directly concern humans and animals, such as infectious diseases, foodborne illnesses and antimicrobial resistance. Comprehensive research in these areas is crucial to combat health challenges within a One Health approach. However, One Health is also a tool to inform policy-makers, to manage infectious disease outbreaks, to implement strategies and to enhance institutionalisation [14]. The current COIVID-19 pandemic has made it painstakingly clear that attention to these broader sets of socio-economic issues are essential in public health responses. Scholars have long expressed concern of silo research in One Health, advocating for more interdisciplinary research to include diverse perspectives (e.g. social, political, anthropological) [17,[27], [28], [29], [30]]. The network analysis indicates that there is a general lack of journals for the type of interdisciplinary research that is promoted by the One Health approach. The network analysis does identify some interdisciplinary journals well-positioned to capture broader socio-economic and management perspectives such as *Social Science & Medicine* and *Ecohealth*. Interestingly, the bibliometric analysis did not reveal One Health research in monodisciplinary social science outlets such as political science, global governance or public administration. Neither did it reveal dedicated One Health outlets. There are a few established One Health journals such as *One Health Outlook* published by BioMed Central and the present *One Health* outlet published by Elsevier for the International Federation for Tropical Medicine. However, these journals are recently established and *One Health Outlook* has not yet made it into the WoS. Stronger cross-sectoral and interdisciplinary One Health research can be promoted by either expanding the thematic areas in existing journals or by increasing the engagement of other types of journals such as those dedicated to One Health or interdisciplinary journals. One Health journals can provide a platform that encourages holistic research from multiple angles, combining quantitative and qualitative research that investigates One Health issues not only as medical and biological themes, but as political as well as socio-cultural themes. Social and political contributions can foster One Health institutionalisation and facilitate policy dialogue. However, the journal network reveals few journals that bridge not just disciplines but whole research traditions, most notably between the medical and social sciences. Hence, interdisciplinary work should be encouraged, as it can promote collaboration, communication as well as knowledge sharing across scientific traditions, disciplines and sectors. One Health and broader public health outlets can facilitate the understanding of complex problems and promote the development of innovations also in the fields of implementation, management, strategy or institutionalisation. Interestingly, absent in our bibliometric analysis were many of the top-tier medical and public health journals, which suggests that One Health research is mainly being published outside the most prestigious international outlets. Indeed, of the 44,063 pieces that have been published in the top ten medical journals in the WoS during the last five years (e.g. *Lancet*, *New England Journal of Medicine*, *British Medical Journal* and *PLOS Medicine*), only six referred to the One Health concept in the title, abstract or keywords. Even among the top ten ranked journals in public, environmental and occupational health (e.g. *Lancet Global Health*, *Lancet Public Health*, *Bulletin of the World Health Organization* and *Annual Review of Public Health*), only seven contributions referred to the One Health concept out a total of 7819 contributions. This modest attention to One Health from the top-tier public health and medical outlets contrasts with the relevance placed on the concept from health practitioners and agencies. The weak academic infrastructure for One Health research risks reproducing a vicious cycle that disincentives new research into One Health due to the more moderate impact factor options available as well as limits the reach and influence of published One Health research.

### 4.2 Themes and interfaces

One of the defining features of a One Health approach is the attention paid to the nexus between human, animal and environment. However, the field of environment is often disregarded in much One Health research. The colour coding of the co-citation networks of journals and authors reveal that environmental perspectives are dwarfed in comparison to epidemiological, microbiological and public health perspectives. Additionally, the co-occurrence of keywords shows that keywords relating to environment, ecology and biodiversity are scarce. These finding are in line with Khan et al. and Lebov et al. who both found that perspectives from the environmental and ecological sector have been neglected within One Health research [30,31]. Further, the co-occurrence network of keywords illustrated that research into One Health is mainly undertaken in the medical science cluster with the most connections to the other clusters. This indicates that a majority of articles is constructed around medical themes, and that there is most interdisciplinary research across areas in the medical science cluster. However, few keywords indicate research into administrative or anthropological approaches to examine the management of One Health. Making these thematic perspectives more central to the network could strengthen the One Health approach regarding implementation and institutionalisation. One Health initiatives and projects that specifically promote mixed methods studies and engage researchers with various expertise could facilitate implementing comprehensive initiatives. Here, a gap in the One Health research could be addressed, facilitating not only quantitative but a qualitative research to comprehensively approach the multifaceted issues implied in One Health topics [32].

There is no shortage of existing outlets, frameworks and approaches that promote interdisciplinary research. Already in 2008, a strategic framework was developed by the tripartite collaborators, as well as the UN System Influenza Coordination, UNICEF and the World Bank, outlining approaches for collaboration, to prevent crises, to govern disease control and surveillance programmes [8]. Rüegg et al. developed a handbook to adapt, improve and optimise One Health activities could also provide some guidance on how to strengthen future One Health activities and evaluate already ongoing One Health initiatives [21]. Coker et al. produced a conceptual framework for One Health, which can be used to develop a strong research strategy to inform policy-making [19]. Lebov et al. have also devised concrete planning guidelines for One Health researchers on how to construct an interdisciplinary and holistic study design that covers all three health domains [30]. Further, guidance documents such as the 2019 published tripartite guide should be considered when implementing One Health activities [2].

### 4.3 Scholars and institutions

The study reveals a high degree of author proximity within and across departments and universities. The physical and academic closeness of the most active scholars might indicate the presence of homophily. Homophily is the tendency of individuals to associate and interact with other individuals similar to them [33]. The proximity might increase effectiveness and create synergies, but risks resulting in a lack of diversity in approaches and themes [34]. Some of these themes might be the environmental issues or social science perspectives.

The citation network also illustrates the centrality of organisations such as the WHO, FAO, CDC, OiE and the World Bank. These organisations appear to have a key role in scientific communication. The organisations have been working together, sharing information, which pushed forward the One Health approach and contributed to the recognition of the approach. This is illustrated by the increase in publications of One Health articles after their engagement in 2010. Especially the WHO is co-cited heavily by various authors and institutes, which is reflective of the institute's engagement with research and guidance on One Health related topics. However, these international organisations appear to completely dominate the policy-cluster at the expense of academic scholars. Thus, there is clearly an opportunity for academic scholars to engage more with the policy field of management, implementation, strategies and policy collaboration in the context of One Health. Furthermore, to facilitate interdisciplinary collaboration and to strengthen the engagement of the environment field into One Health, the FAO/OiE/WHO collaboration could involve the environmental sector. For example, the United Nations Environment Programme could be engaged to push forward and connect human and animal health to the environment. Maybe the tripartite could evolve to a quadripartite agreement? The co-citation network for the ECDC, EFSA and CDC indicates that although they all contribute to similar research areas, only limited connections between them could be traced in their research. Additionally to the analysis of co-citation networks, an investigation into co-authorship could have further shed light on interactions of authors. Another reason for the limited connections between ECDC, EFSA and CDC is that within scientific articles, authors prefer to quote peer-reviewed scientific articles rather than reports. Nevertheless, the lack of co-citations indicates a potential barrier for cooperation beyond the limits of the own organisation. The organisations share core principles of the One Health approach but appear to work in epistemological and/or institutional silos, as evidenced by limited cross-citations between organisations. To facilitate cross-institutional collaboration on One Health research, more focus could be on activities that not only promote interdisciplinarity but cross-institutional engagements such as hosting One Health workshops with broad participation, establishing cross-organisational research groups and encouraging co-authored research projects. Additionally, more flexible research regulations within sectors and improved coordination of engagement of different actors can strengthen work within and across disciplines [35].

## 5 Conclusion

It is essential to take advantage of the current momentum to advance the One Health approach. The momentum is not only reflected by the rising number of publications relating to One Health literature, but also through zoonotic disease outbreaks becoming more frequent, such as Ebola, Zika virus and the current case of COVID-19. The bibliometric analysis showed the potential and increasing interest for One Health. However, it also revealed little engagement with the environmental sector. It indicated that there is a need for more applicable approaches to strengthen intersectoral collaboration and knowledge sharing. Engaging researchers with different expertise and disciplinary backgrounds will facilitate a more comprehensive perspective where One Health is researched in an interdisciplinary way that conceives of the human-animal-environment interface not as separate entities but as a coherent whole. Existing frameworks and guidelines should be used to promote One Health activities. Further, journals dedicated to One Health or interdisciplinary research provide scholars the possibility to publish multifaceted research. Journals, such as *One Health* and *One Health Outlook*, are uniquely positioned to bridge between fields and strengthen interdisciplinary research. With case studies of One Health implementation and themes of governance as well as interdisciplinary collaboration, the journals can also create room for social science approaches alongside of medical and natural sciences.

Despite the success of One Health, there is a need to pay attention to the persistent challenges of integrating social science disciplines, the environmental sector and researchers from diverse disciplines. Nevertheless, the One Health approach has the potential to be established as a comprehensive research field, engaging multifaceted expertise across disciplines.

## Funding statement

This work was supported by the Independent Research Fund Denmark for ‘exploring the policy dynamics of global antimicrobial resistance initiatives’ [grant number 8019-00005B]; and the One Health European Joint Programme (OHEJP) for Work Package 7 ‘Sustainability’ [grant number 773830].

## Declaration of Competing Interest

The authors report no conflicts of interest.

## References

[1] Frazzoli C., Mantovani A. (2019). The Environment-Animal-Human Web: A “One Health” View of Toxicological Risk Analysis [Internet]. https://www.frontiersin.org/research-topics/3593/the-environment-animal-human-web-a-one-health-view-of-toxicological-risk-analysis.

[2] FAO, OiE, WHO (2019). Taking a Multisectoral One Health Approach: A Tripartite Guide to Addressing Zoonotic Diseases in Countries.

[3] Schaer P. (2013). Applied informetrics for digital libraries: an overview of foundations, problems and current approaches. Hist. Soc. Res..

[4] Alonso Aguirre A., Basu N., Kahn L.H., Morin X.K., Echaubard P., Wilcox B.A. (2019 Feb). Transdisciplinary and social-ecological health frameworks-novel approaches to emerging parasitic and vector-borne diseases. Parasit. Epidemiol. Control.

[5] Friese C., Nuyts N. (2017). Posthumanist critique and human health: how nonhumans (could) figure in public health research. Crit. Public Health.

[6] Ward J.M., Meyerholz D.K. (2018). Citation index is not critically important to veterinary pathology, medicine, and research. Vet. Pathol..

[7] Clarivate (2020). Web of Science [Internet]. Web of Science Group. https://clarivate.com/webofsciencegroup/solutions/web-of-science/.

[8] Food and Agriculture Organization, World Organization for Animal Health, World Health Organization, UN System Influenza Coordination, UNICEF, World Bank (2008 Oct). Contributing to One World, One Health - A Strategic Framework for Reducing Risks of Infectious Diseases at the Animal–Human–Ecosystems Interface [Internet]. http://www.fao.org/3/aj137e/aj137e00.pdf.

[9] Leydesdorff L. (2008). On the normalization and visualization of author co-citation data: Salton's cosine versus the Jaccard index. J. Am. Soc. Inf. Sci. Technol..

[10] Hamers L., Hemeryck Y., Herweyers G., Janssen M., Keters H., Rousseau R. (1989 May 1). Similarity measures in scientometric research: the Jaccard index versus Salton's cosine formula. Inf. Process. Manag..

[11] Zhang J., Yu Q., Zheng F., Long C., Lu Z., Duan Z. (2016). Comparing keywords plus of WOS and author keywords: a case study of patient adherence research. J. Assoc. Inf. Sci. Technol..

[12] Blondel V., Guillaume J.-L., Lambiotte R. (2008). Lefebvre E. Fast unfolding of communities in large networks. J. Stat. Mech..

[13] Freeman L.C. (1978 Jan 1). Centrality in social networks conceptual clarification. Soc. Networks.

[14] FAO, OiE, WHO (2010). The FAO-OIE-WHO Collaboration - Sharing Responsibilities and Coordinating Global Activities to Address Health Risks at the Animal-Human-Ecosystems Interfaces - A Tripartite Concept Note [Internet]. https://www.oie.int/fileadmin/Home/eng/Current_Scientific_Issues/docs/pdf/FINAL_CONCEPT_NOTE_Hanoi.pdf.

[15] CDC (2019). 2014–2016 Ebola Outbreak Distribution in West Africa [Internet]. Ebola (Ebola Virus Disease). https://www.cdc.gov/vhf/ebola/history/2014-2016-outbreak/distribution-map.html.

[16] CDC (2017). Reporting and Surveillance - Zika Virus [Internet]. Zike Virus. http://www.cdc.gov/zika/reporting/index.html.

[17] Zinsstag J., Schelling E., Waltner-Toews D., Tanner M. (2011 Sep 1). From “one medicine” to “one health” and systemic approaches to health and well-being. Prev. Vet. Med..

[18] Hitziger M., Esposito R., Canali M., Aragrande M., Häsler B., Rüegg S.R. (2018). Knowledge integration in One Health policy formulation, implementation and evaluation. Bull. World Health Organ..

[19] Coker R., Rushton J., Mounier-Jack S., Karimuribo E., Lutumba P., Kambarage D. (2011 Apr). Towards a conceptual framework to support one-health research for policy on emerging zoonoses. Lancet Infect. Dis..

[20] Swiss TPH. Swiss TPH (2020). Swiss Tropical and Public Health Institute [Internet]. Swiss TPH. https://www.swisstph.ch/en/.

[21] Rüegg S.R., Häsler B., Zinsstag J. (2018). Integrated Approaches to Health: A Handbook for the Evaluation of One Health [Internet].

[22] Haesler B., Cornelsen L., Bennani H., Rushton J. (2014). A review of the metrics for one health benefits. Rev. Sci. Tech..

[23] Rüegg S.R., McMahon B.J., Häsler B., Esposito R., Nielsen L.R., Ifejika Speranza C. (2017). A Blueprint to Evaluate One Health. Front Public Health [Internet].

[24] EFSA (2016 Apr). EFSA Strategy 2020 Trusted Science for safe Food [Internet]. https://scar-europe.org/images/SCAR-Documents/efsa_strategy2020.pdf.

[25] EFSA, ECDC (2019). The European Union One Health 2018 zoonoses report. EFSA J..

[26] EFSA, ECDC (2019). The European Union summary report on antimicrobial resistance in zoonotic and indicator bacteria from humans, animals and food in 2017. EFSA J..

[27] Degeling C., Johnson J., Kerridge I., Wilson A., Ward M., Stewart C. (2015 Dec 29). Implementing a One Health approach to emerging infectious disease: reflections on the socio-political, ethical and legal dimensions. BMC Public Health.

[28] dos Ribeiro S.C., LHM Van De Burgwal, Regeer B.J. (2019 Jun 1). Overcoming challenges for designing and implementing the One Health approach: a systematic review of the literature. One Health.

[29] Manlove K.R., Walker J.G., Craft M.E., Huyvaert K.P., Joseph M.B., Miller R.S. (2016 Apr). ‘One Health’ or Three? Publication Silos Among the One Health Disciplines. PLoS Biology; San Francisco [Internet]. http://search.proquest.com/docview/1789551398/abstract/183871A71C4E4F17PQ/1.

[30] Lebov J., Grieger K., Womack D., Zaccaro D., Whitehead N., Kowalcyk B. (2017 Jun 1). A framework for One Health research. One Health.

[31] Khan M.S., Rothman-Ostrow P., Spencer J., Hasan N., Sabirovic M., Rahman-Shepherd A. (2018 Jun 1). The growth and strategic functioning of One Health networks: a systematic analysis. Lancet Planet. Health.

[32] Degeling C., Rock M. (2020). Qualitative research for one health: from methodological principles to impactful applications. Front. Vet. Sci..

[33] McPherson M., Smith-Lovin L., Cook J.M. (2001). Birds of a feather: homophily in social networks. Annu. Rev. Sociol..

[34] Stoica A.-A. (2018 Dec 1). Homophily in co-autorship networks. Int. Rev. Soc. Res..

[35] Fletcher I., Birko S., Dove E.S., Laurie G.T., McMillan C., Postan E. (2020 Jun 1). Co-production and managing uncertainty in health research regulation: a Delphi study. Health Care Anal..

